# Hepatic Sarcoidosis: A Case Report and Literature Review

**DOI:** 10.7759/cureus.71873

**Published:** 2024-10-19

**Authors:** Eli A Zaher, Mohamed A Ebrahim, Parth Patel

**Affiliations:** 1 Internal Medicine, Ascension Saint Joseph Hospital, Chicago, USA

**Keywords:** angiotensin converting enzyme, granulomas, non-caseating granulomas, pruritus, sarcoidosis

## Abstract

Sarcoidosis is a granulomatous disease that can affect multiple organs, including the liver. We present a case of a 43-year-old male with hepatic sarcoidosis who presented with jaundice and pruritus. Laboratory findings showed mixed hyperbilirubinemia and elevated liver enzymes. Imaging revealed mediastinal lymphadenopathy and diffuse liver changes, raising suspicion for sarcoidosis. Elevated angiotensin-converting enzyme (ACE) levels and a liver biopsy confirmed the diagnosis. The patient was treated with ursodeoxycholic acid, leading to symptomatic improvement. This case highlights the importance of recognizing hepatic involvement in sarcoidosis and the potential role of ursodeoxycholic acid in its management.

## Introduction

Sarcoidosis is an inflammatory disease of uncertain cause, characterized by non-caseating granulomas that affect various organs. Its global prevalence ranges from two to 60 per 100,000 people. It is more frequent in women, regardless of ethnicity. In the U.S., African Americans have a threefold higher incidence [1]. 

Epithelioid granulomas, typically non-caseating, are the hallmark of sarcoidosis, with the lungs involved in about 90% of cases. Around 40%-50% of patients also experience extra-thoracic sarcoidosis, affecting lymph nodes, liver, spleen, gastrointestinal tract, bones, and skin. Solid organ involvement can cause enlargement, while dermatological manifestations include erythema nodosum and lupus pernio. Less common symptoms include myopathy, uveitis, granulomatous meningitis, and facial nerve palsy [2]. 

Sarcoidosis presents in two main syndromes: Löfgren syndrome, marked by hilar lymphadenopathy, erythema nodosum, arthritis, and fever, and Heerfordt syndrome, characterized by uveitis, parotid swelling, fever, and facial palsy [3]. 

The disease's pathogenesis is thought to involve a mix of immune, genetic, and environmental factors. An environmental trigger may initiate an immune response in genetically predisposed individuals, with T-helper-1 cells and cytokines playing key roles in granuloma formation. Possible triggers include viruses and bacteria [2-4]. 

We present a case of hepatic sarcoidosis in a young male with a rare initial presentation, jaundice, and pruritus.

## Case presentation

A 43-year-old male with a history of anxiety presented to the emergency room due to two weeks of generalized body aches, pruritus, and jaundice. He had never experienced such symptoms before. He denied any abdominal pain, swelling, fevers, chills, nausea, vomiting, weight loss, diarrhea, constipation, or bloody or dark stools. He denied using any prescription or over-the-counter medications. He also denied using alcohol, intravenous drugs, or smoking. 

Physical examination showed scleral icterus and generalized jaundice without swelling, hepatomegaly, splenomegaly, or ascites. Admission vitals were within normal limits. Laboratory workup upon admission revealed normocytic anemia and mixed hyperbilirubinemia (Table 1). 

**Table 1:** The patient's blood work upon admission

Component	Result	Reference range
Hemoglobin (g/dL)	11.0	12.0-15.3
White cell count (k/mm cu)	8.0	4.0-11.0
Mean corpuscular volume (f/L)	92	80.0-100.0
Platelets (k/mm cu)	279	150-450
Creatinine (mg/dL)	0.5	0.6-1.2
Sodium (mmol/L)	130	133-144
Albumin (g/dL)	2.9	3.5-5.7
Aspartate aminotransferase (IU/L)	201	13-39
Alanine aminotransferase (IU/L)	149	7-52
Total bilirubin (mg/dL)	3.0	0.0-1.0
Direct bilirubin (mg/dL)	1.5	<0.3
Alkaline phosphatase (IU/L)	386	35-104
International normalized ratio	1.0	0.9-1.1
Ferritin (ng/mL)	1313	24-336
Iron (ug/dL)	204	50-212
Lactate dehydrogenase (U/L)	242	140-280

**Figure 1:**
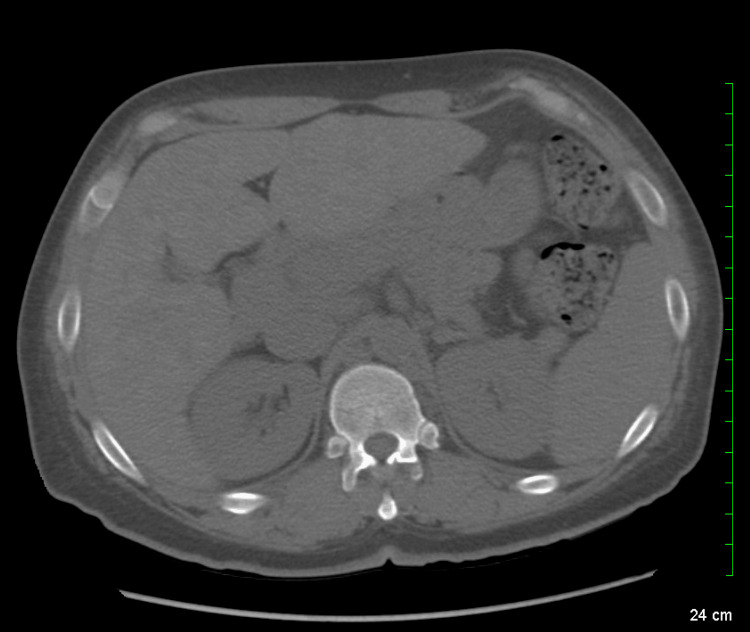
A CT scan of the abdomen without contrast; diffuse heterogenous attenuation of the liver is noted.

**Figure 2:**
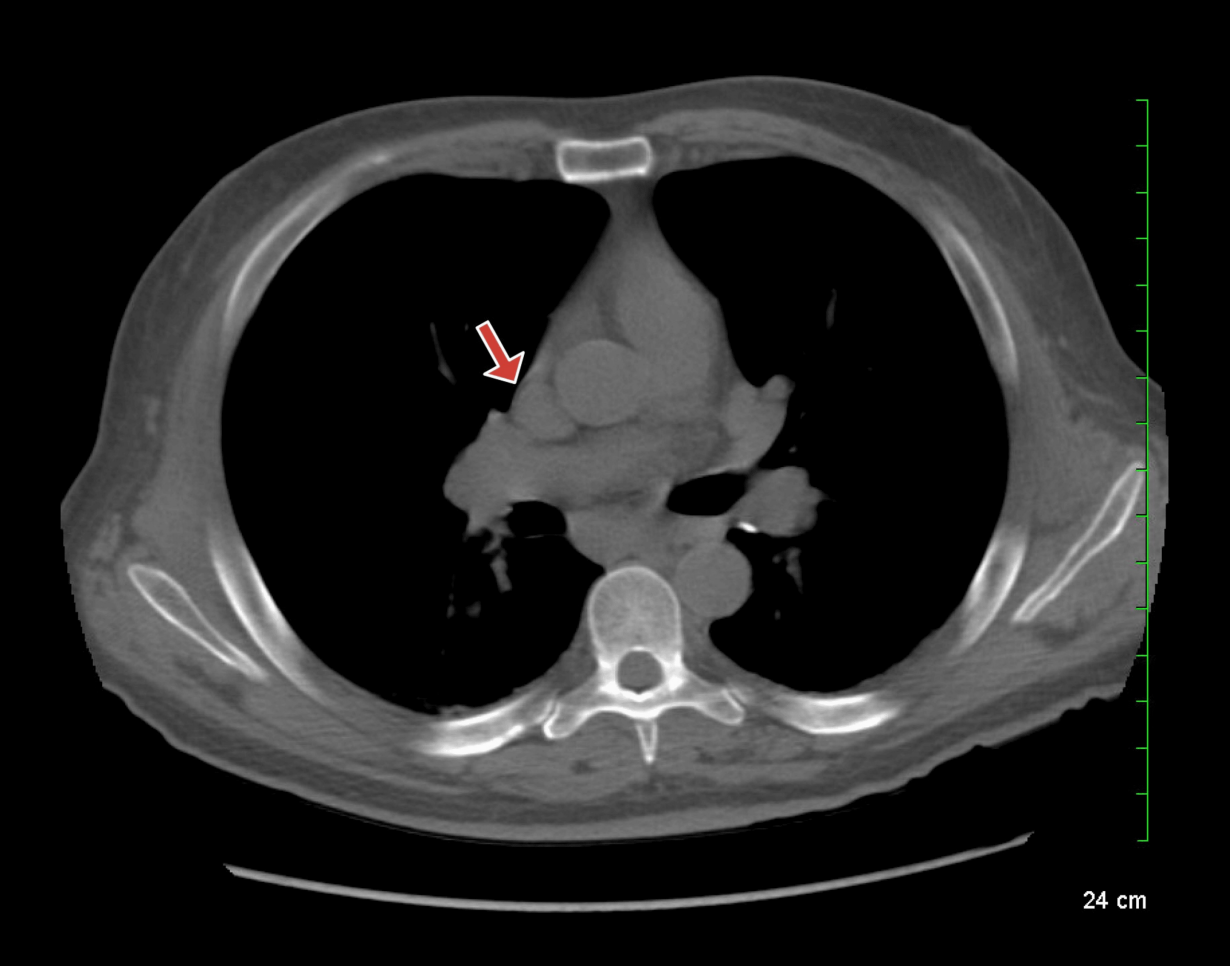
A CT scan of the chest without contrast; mediastinal lymphadenopathy is noted (red arrow).

**Figure 3:**
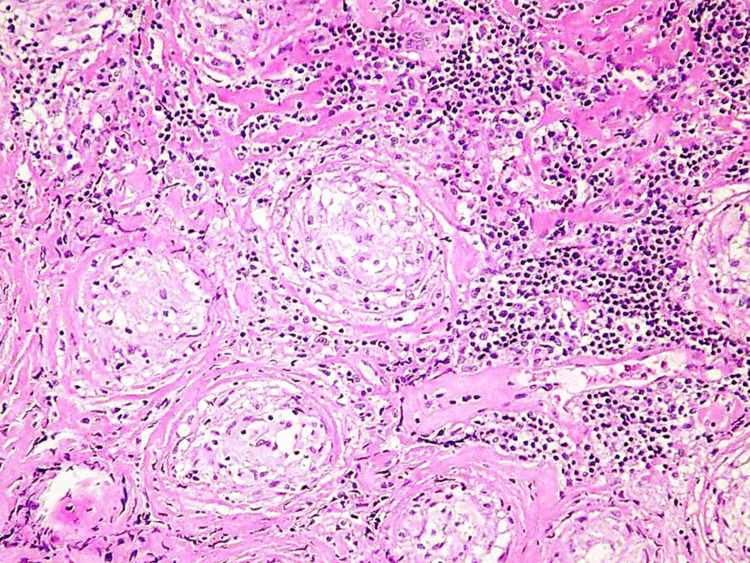
The liver biopsy shows non-caseating epithelioid granulomas Non-caseating granulomas are characterized by compact clusters of epithelioid histiocytes and multi-nucleated giant cells, distinctly lacking central necrosis. They are prominently located near the portal tracts and extend into the lobular parenchyma.

Computed tomography (CT) was done without contrast due to allergy and revealed a diffuse heterogeneous attenuation of the liver and bilateral mediastinal lymph node enlargement, raising suspicion for sarcoidosis (Figures 1-2). Levels of angiotensin-converting enzyme (ACE) were thus collected and were elevated to 176 U/L (reference range, 9-67 U/L). Gastroenterology was consulted and recommended magnetic resonance imaging (MRI) of the abdomen, but the patient was unable to tolerate it due to severe claustrophobia. An ultrasound-guided percutaneous core liver biopsy was done with results consistent with sarcoidosis (Figure 3). 

The patient was started on ursodeoxycholic acid and discharged home with outpatient follow-up.

## Discussion

Sarcoidosis is an inflammatory disease of uncertain cause, characterized by non-caseating granulomas that affect various organs. It occurs without infections, other autoimmune conditions, or exposure to foreign substances. Globally, the prevalence of sarcoidosis is estimated to range from two to 60 cases per 100,000 people. The disease affects all ethnic groups, with a higher prevalence seen in individuals of Scandinavian origin. In contrast, it is rarely reported in those of Chinese and Taiwanese descent. In the U.S., African Americans have been shown to have an incidence rate three times higher than the age-adjusted average [1, 2].

The exact cause of sarcoidosis remains unclear, but it is believed to result from a combination of immunological, genetic, and environmental factors [3]. One hypothesis suggests that an environmental trigger activates a specific immune response in individuals with a genetic predisposition. In sarcoidosis, this immune response involves T helper 1 (Th1) cells reacting to an antigen, which leads to the production of cytokines, primarily tumor necrosis factor-alpha. This in turn causes macrophages to aggregate and form granulomas [5]. Certain histocompatibility antigens, such as HLA-A1, B8, DRB1, DQB1, and DRB3, have been linked to the disease, indicating a genetic susceptibility and familial clustering [4]. Potential environmental triggers may include reactive oxygen species, as well as viruses like herpes simplex, cytomegalovirus, retroviruses, and bacteria such as *Borrelia burgdorferi* and mycobacteria [2].

Liver involvement in sarcoidosis can present in a variety of ways. While over 50% of patients show hepatic granulomas on liver biopsy, only 10%-30% have elevated liver enzymes [6]. About one in five patients may have hepatomegaly or splenomegaly. Imaging techniques such as ultrasound or CT are effective in detecting organ enlargement, identifying it in about 40% of cases [7]. However, even when serum liver enzymes are elevated or radiological abnormalities are present, actual organ dysfunction is uncommon. Certain clinical symptoms may suggest hepatic involvement and indicate the need for a liver biopsy. General symptoms like fatigue, fever, and joint pain are non-specific but occur in most patients with active liver sarcoidosis. More specific signs include jaundice and itching, often resulting from chronic cholestasis. Right upper quadrant pain, thought to be due to hepatomegaly stretching the Glisson’s capsule as granulomas enlarge within the liver, occurs in 15% of cases, while jaundice is reported in fewer than 5% of patients [8, 9].

Corticosteroids have been shown to reduce the number of hepatic granulomas by suppressing the inflammatory response and decreasing liver size, which may alleviate abdominal pain caused by hepatomegaly. Steroids are also recommended for patients with systemic symptoms such as fever, fatigue, itching, and weight loss. Treatment duration depends on clinical response, with some recommending a 12-month course before tapering. Long-term therapy may be required for relapsing symptoms, and steroid-sparing agents could be considered [10]. In contrast, ursodeoxycholic acid reduces the biliary secretion of certain bile acids and inhibits the intestinal absorption of bile salts. It also modulates the immune response by decreasing HLA class I and II antigens on liver and bile duct cells. Recent studies suggest that this drug is beneficial in treating hepatic sarcoidosis, particularly in patients with symptomatic cholestasis such as pruritus. It may also slow disease progression [11]. Due to its safety profile compared to steroids, it is recommended to use ursodeoxycholic acid as a first-line treatment. In a study comparing placebo, prednisone, and ursodeoxycholic acid, the latter showed superior improvement in liver enzyme levels, pruritus, and fatigue [12]. Second- and third-line agents may be helpful for patients who require but do not respond to prednisone or become dependent on steroids. Azathioprine has been shown to normalize liver enzymes but can also lead to acute hepatitis. Other drugs, such as methotrexate, glutathione, chlorambucil, cyclosporine, cyclophosphamide, thalidomide, pentoxifylline, and infliximab, have been reported to offer benefits, though there is insufficient evidence to fully support their use [11, 12].

## Conclusions

We present a case of systemic sarcoidosis with primary liver involvement, where the main symptoms were generalized pruritus and jaundice. Liver biopsies are crucial for diagnosing and monitoring liver sarcoidosis, as many patients exhibit epithelioid non-caseating granulomas, while others may have hepatomegaly or abnormal liver enzyme levels. Ursodeoxycholic acid has been shown to significantly alleviate symptoms and may slow the progression of the disease. Given its favorable safety profile, starting treatment with ursodeoxycholic acid is recommended. However, further research is needed to clarify the specific role of ursodeoxycholic acid in managing hepatic sarcoidosis by investigating its long-term effectiveness, optimal dosing, and its potential to halt disease progression. Studies comparing ursodeoxycholic acid with other treatment options and evaluating its use in various stages of hepatic sarcoidosis would also help establish clearer treatment guidelines.

## References

[1] Rybicki BA, Major M, Popovich J Jr, Maliarik MJ, Iannuzzi MC (1997). Racial differences in sarcoidosis incidence: a 5-year study in a health maintenance organization. Am J Epidemiol.

[2] Iannuzzi MC, Rybicki BA, Teirstein AS (2007). Sarcoidosis. N Engl J Med.

[3] Culver DA (2012). Sarcoidosis. Immunol Allergy Clin North Am.

[4] Lazarus A (2009). Sarcoidosis: epidemiology, etiology, pathogenesis, and genetics. Dis Mon.

[5] Zissel G, Prasse A, Müller-Quernheim J (2010). Immunologic response of sarcoidosis. Semin Respir Crit Care Med.

[6] Chen ES, Moller DR (2013). Sarcoidosis. CURRENT Diagnosis & Treatment: Rheumatology, 3e.

[7] Ebert EC, Kierson M, Hagspiel KD (2008). Gastrointestinal and hepatic manifestations of sarcoidosis. Am J Gastroenterol.

[8] Harder H, Büchler MW, Fröhlich B, Ströbel P, Bergmann F, Neff W, Singer MV (2007). Extrapulmonary sarcoidosis of liver and pancreas: a case report and review of literature. World J Gastroenterol.

[9] Blich M, Edoute Y (2004). Clinical manifestations of sarcoid liver disease. J Gastroenterol Hepatol.

[10] Moller DR (2003). Treatment of sarcoidosis - from a basic science point of view. J Intern Med.

[11] Cremers JP, Drent M, Baughman RP, Wijnen PA, Koek GH (2012). Therapeutic approach of hepatic sarcoidosis. Curr Opin Pulm Med.

[12] Bakker GJ, Haan YC, Maillette de Buy Wenniger LJ, Beuers U (2012). Sarcoidosis of the liver: to treat or not to treat?. Neth J Med.

